# Interventionally implanted port catheter systems for hepatic arterial infusion of chemotherapy in patients with colorectal liver metastases: A phase II-study and historical comparison with the surgical approach

**DOI:** 10.1186/1471-2407-7-69

**Published:** 2007-04-24

**Authors:** Bert Hildebrandt, Maciej Pech, Annett Nicolaou, Jan M Langrehr, Jacek Kurcz, Birgit Bartels, Alexandra Miersch, Roland Felix, Peter Neuhaus, Hanno Riess, Bernd Dörken, Jens Ricke

**Affiliations:** 1 CharitéCentrum für Tumormedizin, Medizinische Klinik mit Schwerpunkt Hämatologie und Onkologie, Campus Virchow Klinikum, Charité-Universitätsmedizin Berlin, Augustenburger Platz 1, D-13344 Berlin, Germany; 2 CharitéCentrum für Tumormedizin, Klinik für Strahlenheilkunde, Campus Virchow Klinikum, Charité-Universitätsmedizin Berlin, Augustenburger Platz 1, D-13344 Berlin, Germany; 3 Klinik für Radiologie und Nuklearmedizin, Otto-von-Guericke-Universität Magdeburg, Leipziger Str. 44, D-30120 Magdeburg, Germany; 4 CharitéCentrum für Chirurgische Medizin, Klinik für Allgemein-, Viszeral- und Transplantationschirurgie, Campus Virchow Klinikum, Charité Universitätsmedizin Berlin, Augustenburger Platz 1, D-13344 Berlin, Germany; 5 Akademia Medyczna Katedra i Zakład Radiologii, Szpital Kliniczny Nr 1, M. Skłodowskiej-Curie St. 68, PL-50369 Wrozlaw, Poland

## Abstract

**Background:**

The high complication rates of surgically implanted port catheter systems (SIPCS) represents a major drawback in the treatment of isolated liver neoplasms by hepatic arterial infusion (HAI) of chemotherapy. Interventionally implanted port catheter systems (IIPCS) have evolved into a promising alternative that enable initiation of HAI without laparatomy, but prospective data on this approach are still sparse. Aim of this study was to evaluate the most important technical endpoints associated with the use of IIPCS for the delivery of 5-fluorouracil-based HAI in patients with colorectal liver metastases in a phase 2-study, and to perform a non-randomised comparison with a historical group of patients in which HAI was administered via SIPCS.

**Methods:**

41 patients with isolated liver metastases of colorectal cancer were enrolled into a phase II-study and provided with IIPCS between 2001 and 2004 (group A). The primary objective of the trial was defined as evaluation of device-related complications and port duration. Results were compared with those observed in a pre-defined historical collective of 40 patients treated with HAI via SIPCS at our institution between 1996 and 2000 (group B).

**Results:**

Baseline characteristics were balanced between both groups, except for higher proportions of previous palliative pre-treatment and elevated serum alkaline phosphatase in patients of group A. Implantation of port catheters was successful in all patients of group A, whereas two primary failures were observed in group B. The frequency of device-related complications was similar between both groups, but the secondary failure rate was significantly higher with the use of surgical approach (17% vs. 50%, p < 0.01). Mean port duration was significantly longer in the interventional group (19 vs. 14 months, p = 0.01), with 77 vs. 50% of devices functioning at 12 months (p < 0.01). No unexpected complications were observed in both groups.

**Conclusion:**

HAI via interventionally implanted port catheters can be safely provided to a collective of patients with colorectal liver metastases, including a relevant proportion of preatreated individuals. It appears to offer technical advantages over the surgical approach.

## Background

The role of hepatic arterial infusion in patients with colorectal liver metastases has been frequently studied throughout the past decades. More than a dozen of randomized trials have been completed in which HAI with one of the fluoropyrimidines floxuridine (FUDR) or 5-flourouracil (5-FU) was compared to intravenous drug application in patients with unresectable metastases or after resection (reviewed in [1,2]). In summary, HAI largely improved response rates in patients with unresectable disease and time to hepatic progression for both indications. However, results are counterbalanced by the fact that many of the trials did not reveal an increase in overall survival.

The reasons preventing a clear-cut survival benefit for HAI have been discussed in detail elsewhere [1-6]. Small patient numbers and inappropriate control groups may have led to a misleading interpretations particularly in the earlier trials. In addition, studies on intraarterial FUDR revealed an excessively high rate of extrahepatic failure. Adjacent trials completed in North America demonstrated that systemic control rates of intraarterial FUDR can be improved by combination with intravenous 5-FU [7,8]. In contrast, European and Asian investigators aimed to optimise results by taking the advantage of achieving systemic drug concentrations and limiting hepatic toxicity as it is provided by the intraarterial application of 5-FU [9,10].

Unlike hepatic arterial FUDR – which is usually delivered through surgically implanted infusion pumps – regional applications of 5-FU demand the use of external pumps, due to the maximal drug concentration of 25 mg/ml, and the resulting high volumes of medication. Therefore, hepatic arterial 5-FU can only be delivered by intermittent percutaneous access, or through hepatic arterial port systems. There is profound experience with the use of such port catheters in a number of specialised treatment centres worldwide. However, this technique is associated with a considerable proportion of primary failure which has been reported to approximate one third of patients scheduled for 5-FU-based HAI in recent multicenter trials [9,11]. Once HAI has been started, port systems are associated with a higher complication rate than surgically implanted pumps, whereas secondary failure rates have been reported to be similar (e.g. approximately half of patients within the first 12 months of treatment) [12-14]. Today, it is widely accepted that the low performance level of surgically implanted port catheters represents one major reason why the theoretical advantage of 5-FU-based HAI could not be transferred to convincing clinical improvements so far.

Interventionally implanted port catheter systems (IIPCS) have evolved into a promising alternative to surgically implanted devices. IIPCS enable initiation of HAI without laparatomy, and available data suggest favorable complication and failure rates. However, studies published so far mainly focussed on either technical or clinical aspects of treatment [10,15-20]. Thus it is still uncertain how these devices compare to surgically implanted port catheters.

We here present an evaluation of technical endpoints associated with the use of interventionally implanted hepatic arterial port catheter systems for hepatic arterial infusion of chemotherapy in a group of patients with colorectal liver-only metastases, as well as, a non-randomised comparison with those observed within a historical collective of patients provided with surgically implanted devices.

## Methods

### Study design, patients' collectives and eligibility criteria

Patients with isolated liver metastases of colorectal cancer were prospectively enrolled into a phase II-trial evaluating technical complications associated with the use of interventionally implanted port catheters in patients with cancers confined to the liver (ClinicalTrials.gov Identifier: NCT00356161). Complication rates and duration of interventionally implanted port catheter systems, as well as, reasons for primary and secondary device failure of the first 41 colorectal cancer patients enrolled were assessed, and compared with those observed in a pre-defined [21] historical collective of 40 patients with colorectal liver metastases provided with a surgically implanted port system. The protocol and the evaluation presented herein were approved by the local ethics committee, and a detailed written informed consent was obtained from every patient prior to treatment.

#### Group A: Interventionally implanted ports catheter systems (IIPCS)

Adult patients with histologically proven adenocarcinoma of the colon or rectum, an ECOG performance status of 0–2, and an estimated life expectancy of ≥ 6 months were enrolled into a phase II-trial between March 2001 and May 2004 if they had irresectable liver metastases or underwent hepatic resection or ablation in between the last 8 weeks.

The primary objective was to evaluate the technical complications associated with port implantation and adjacent hepatic arterial infusion of chemotherapy, and of port duration (time to port dysfunction). Secondary endpoints included a comparison of these endpoints to those observed within a historical collective of patients provided with a surgically implanted device, as well as, evaluation of a standardized approach of chemotherapy application according to the different treatment indications, chemotherapy toxicity, response and survival rates.

Patients were excluded if they had a history of secondary cancers in between the last three years, if they suffered from extrahepatic metastases, were previously treated with percutanoeous liver irradiation; had severe restrictions of vital organ function (in particular cardiovascular or liver disease), other severe medical conditions or contraindications against the cytostatic drugs to be applied.

The port implantation procedure has been described in detail by Ricke et al. ([18]). It consisted of the placement of a standard angiography catheter in the hepatic artery and subcutaneous connection to a port system (Titakath; Innovent, Hürth, Germany) placed inferior to the groin by using a titanium connector (Arrow, Reading, PA, USA). Patency and integrity of the system was documented by digital subtraction angiography (DSA) and complemented by a scintigraphy using (99 m)Tc-labelled macroaggregated albumin if necessary. DSA was repeated before each treatment course. In a subset of patients, port patency was additionally assessed by SPECT-CT [22].

#### Group B: Patients with surgically implanted port catheter systems (SIPCS)

Group B consisted of all colorectal cancer patients provided with SIPCS which were assigned to HAI within the scope of a standardised protocol between 1996 and the introduction of IIPCS in 2000. After identifying patients by database query, a retrospective evaluation of medical records was performed with regard to the same endpoints as for patients of group A before the start of above-mentioned phase II trial [21]. Patients included into one multicenter trial were excluded due to the different chemotherapy protocol applied [23]. Three of the patients included had already been communicated as case-reports [24,25].

Implantation of the device was performed in the scope of laparatomy (as described by [9]), with the port chamber being placed subcutaneously overlying the right lower ribs. Total liver perfusion was controlled intraoperatively with the injection of fluorescein dye (5 mL). Postoperatively, patency and integrity of the port system was documented by DSA, complemented by a scintigraphy using (99 m)Tc-labeled macroaggregated albumin if necessary.

### Interventions

#### Hepatic arterial infusion of chemotherapy

Because the patient population was anticipated to be heterogenously with respect to pretreatment and treatment indications, the phase II-protocol on IIPCS allowed different chemotherapy regimen based on the protocol of the German Cooperative Group on Liver Metastases as previously described [18,24]. Patients not pretreated in a palliative intent were assigned to intra-arterial folinic acid (170 mg/m^2^, d1-5, q28) followed by intra-arterial 5-FU (600 mg/m^2^, 120 minutes). The only difference between both groups with regard to chemotherapy was that FA was delivered as a mixture of sodium folinate and 5-FU over 120 min. in group A, and consecutively as calcium folinate over 30 min. followed 5-FU (120 min) in group B. A 5-FU dose escalation of 10% per cycle was performed until the occurrence of adverse reactions (WHO I-II). In patients with hepatic disease progression while undergoing this treatment, regional chemotherapy was escalated by adding mitomycin (8 mg/m^2^, 120 minutes) on day 1 of each cycle. Since July 2002, patients in group A were preferentially switched to weekly FA/5-FU complemented by intraarterial oxaliplatin (50 mg/m^2^, 120 minutes) for 6 of 8 weeks instead, whereas patients with extrahepatic disease progression were scheduled to weekly FA/5-FU complemented by weekly intravenous irinotecan (125 mg/m^2^, 60–90 minutes) for 3 out of 4 weeks. As patients of group A started HAI before the introduction of oxaliplatin and irinotecan, folinic acid and continuous infusional 5-FU at a dose of 2.6–3,6 g/m^2 ^applied over 4–48 h was considered as salvage treatment. The final decision on the chemotherapy schedule was at the physicians' discretion and based on the individual pretreatments.

### Evaluations

#### Pretreatment evaluation and follow-up

Each patients' history was recorded and clinical examination was performed. In patients of group A, this was done prospectively within the 14 days preceding the first chemotherapy application, as well as, haematologic and biochemical laboratory analyses were performed. Imaging procedures comprised an abdominal CT or MRT and chest-x-ray or CT in every patient. Clinical evaluation, a full blood count and clinical chemistry were repeated at least monthly. Assessment of toxicity was performed after every treatment course according to WHO-criteria. In patients of both groups, information on pretreatment and clinical course were extracted from the patients medical records, and supplemented by systematic queries of findings recorded on the databases of the Surgical and Radiological Clinic.

Response evaluation according to WHO criteria and evaluation of tumor markers were repeated at least every 3 months during treatment. After completion of therapy, patients were followed up by clinical investigations every 3 months, complemented by response evaluation until disease progression, initiation of salvage treatment or death. The efficacy analyses included objective response rates, as well as, progression free- and overall survival.

Treatment was interrupted if irreversible loss of function of the port device, WHO toxicity IV° (except for haematotoxicity), or disease progression after escalation of treatment occurred. The dosage of cytostatics was reduced if WHO-toxicity III° or haematotoxicity III/IV° appeared in between of two treatment courses or toxicity >WHO I° was ongoing on the first day of the following cycle (except for leukocytopenia: >WHO II° and thrombocytopenia: >II°). For combination regimens, it was at the physicians discretion to reduce all or only one dosage of the compounds.

Due to the standardized pretreatment assessment of patients provided with surgically implanted port catheters, the retrospective evaluation of the patients' record, documentation, and analyses of endpoints of patients of group B were largely the same as for group A. Data analyses had already been completed before the initiation of the phase II-trial [21].

#### Port complications, port duration, and toxicity

Device-related adverse events were assessed from the time point of successful implantation. Patency and integrity of the port system was documented by DSA performed before every treatment course, complemented by a scintigraphy of the liver with Tc-99 m labeled macroaggregated albumine when gross pathological findings were observed. Port duration was defined as functional device with or without revision, but without need for complete system explantation. Patients in which HAI was stopped because of disease progression were censored at the last application of HAI.

#### Statistical evaluations

Differences between proportions were analyzed by chi-square tests. Mann-Whitney tests were used to compare quantitative and ordinal variables. Univariate analysis of port duration was calculated according to the Kaplan-Meier method, and comparisons between groups were calculated by Log-Rank tests. A two-sided p-value less than 0.05 was considered to indicate significance for all tests. All analyses were performed by using the SPSS 12.0 software package.

## Results

### Patients' and treatment characteristics

Baseline characteristics were similar between patients enrolled into group A and those of group B. However, higher rates of palliative pretreatment and elevated serum alkaline phosphatase indicate a rather unfavorable prognosis for patients of group A (Table 1).

The proportions of patients with nonresectable liver metastases and those who had undergone hepatic resection or hepatic ablation procedures within the past 8 weeks (and thus were treated in an "adjuvant" intent) were comparable between groups A and B (Table 2). Port implantation was successful and regional therapy was started in all (n = 41) patients of group A (100%), whereas primary port failure due to hepatic artery thrombosis was recorded in two out of 40 patients of group B. The rate of patients starting HAI as combination chemotherapy was significantly higher in patients of group A than of group B. The same held true for patients who received either oxaliplatin or irinotecan during their course of treatment (Table 2).

Revisions of the port system were required in 23 patients of group A, and were all carried out in an interventional way. Thereby, a dislocation of the catheter tip was the most common reason for first revision (n = 8), followed by leakage/extravasation (n = 5), thrombus/embolisation (n = 5), disconnection of catheter and port chamber (n = 3), catheter occlusion (n = 1), port chamber infection (n = 1). In group B, a surgical revision of the device was performed in 4 patients, due to rotation (n = 2)/dislocation (n = 1) of the port chamber, or dislocation of catheter tip (n = 1). Taken together, the number of revisions were more frequent in group A than in group B, whereas the rates of patients requiring local thrombolytic therapy were comparable.

The numbers of patients who received six courses of folinic acid and 5-FU in an adjuvant intent were similar in both groups (8/10 in group A vs. 7/9 pts. in group B). All eight patients of group A had a functioning regional chemotherapy device at post-therapeutic restaging, whereas port failure was diagnosed immediately after completion of adjuvant chemotherapy in three additional patients of group B. At a mean observation time of 8.0 vs. 8.4 months (p = 0.73), the proportion of patients of group A who regularly completed treatment with an intact regional chemotherapy device, or had a functioning port system at the end of the study, was significantly higher than in group B. In addition, the rate of patients in which HAI had to be terminated due to terminal device dysfunction was significantly lower (Table 2).

#### Comparison of port complications and port duration

In group A, a total of 39 device-related complications were recorded in 26 patients (63%). Those mostly comprised vascular events such as dislocation of the catheter tip, or thrombosis of the hepatic artery or one of its branches. 9 patients experienced more than one complication. In group B, the respective rates were only slightly higher (tables 2 and 3).

Complications of vascular origin were less common in group A than in group B, although dislocations of the catheter tip were more frequently observed in group A (table 3). However, the latter could be regularly revised, and the incidence of hepatic arterial thrombosis was lower than in group B. In addition, hepatic arterial stenosis (defined as angiographically detected stenosis without thrombotic material or signs of dissection at the catheter tip) was exclusively observed in 7 patients of group B (table 3). The higher vascular complication rate in group B translated into a significant higher proportion of treatment-limiting vascular complications (table 4).

**Figure 1:**
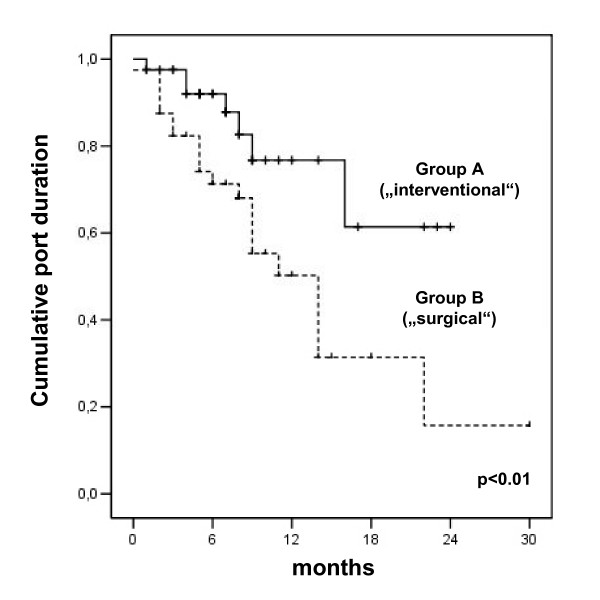
Comparison of port duration between interventionally and surgically implanted devices.

Rates of patients with functional devices at 12 months were 77 vs. 50% (p < 0.01). The mean port duration was 19 months for patients in group A and 14 months for patients of group B (log-rank p = 0.01) (figure 1).

#### Chemotherapy toxicity

WHO grade 3 adverse events were observed in patients of group A/B as follows: Nausea/emesis 6/3, mucositis 6/3, Diarrhoe 7/5, anemia 1/0, leucocytopenia 7/2, thrombocytopenia 1/0. Episodes of grade 4 toxicity comprised mucositis 1/0 and thrombocytopenia 1/0, anemia 1/0. Severe abdominal pain occurred in 4 patients of group A. Gastric or duodenal ulcer due to treatment-related abdominal pain was diagnosed in 1/2 patients. No severe hepatic toxicity was observed.

## Discussion

Up to now, only few reports on the use of IIPCS in any pre-defined tumor entity or treatment indication are available. Indeed, studies dealing with technical considerations in detail mostly evaluated patients with different cancers (reviewed by Heinrich et al [14]), whereas studies evaluating novel HAI-approaches for colorectal cancer patients did not give a detailed description on technical aspects [10,17]. With this report, we give a detailed description on the technical aspects of HAI provided via IIPCS in patients with colorectal liver metastases for the first time.

Probably most important, we demonstrated that all patients scheduled for HAI via an interventionally implanted port catheter could be provided with a functioning device. The virtual absence of primary device failure with the use of IIPCS has been described earlier by others (reviewed in [14]) and us [18]. However, it is remarkable that we were able to reproduce such a result in a prospective manner in a collective of patients with colorectal liver metastases in which 41% of patients were pre-treated by hepatic resection or ablation, and in which the same percentage had previously received at least one palliative line of chemotherapy.

The rate of catheter-associated complications observed with the use of interventionally implanted port devices has been reported to range from 4–56% [14]. In the largest trial on IIPCS available on so far, the authors calculated a rate of 25% of device failures, but did not add a detailed description of actual complications to their report [10]. In the present study, the rate of patients experiencing at least one device-related complication was considerably higher (63%), but a high proportion of events could be successfully corrected by interventional revisions. This particular holds true for dislocations of the catheter tip and complications affecting the connection of the catheter and port chamber which did not result in terminal device failure in any of the patients affected. In summary, our rate of 17% device-related treatment interruptions with the use of interventionally implanted port catheters compares favourable with most previous publications.

In a larger perspective, results achieved with the use of IIPCS have to compare with those observed with surgically implanted devices. Therefore, a substantial part of our presentation deals with the comparison of technical endpoints between the IIPCS and SIPCS for the delivery of 5-fluorouracil-based HAI in patients with colorectal liver metastases. For SIPCS, previous studies have reported major complication rates in the range of 22–48%, and port durations of 7–13 months [12-14]. In our SIPCS group, the mean device duration of 14 months compares favourable with this data, despite the relatively high failure rate of secondary device failure.

The direct comparison of device-related complications did not reveal significant differences between our group of patients on the first sight. However, there was a trend towards a higher rate of revisions in the interventional group which was mostly due to dislocations of the catheter tip and non-vascular events. Those catheter dislocations are mostly manageable from a technical point of view, but one has to keep in mind that extrahepatic delivery of chemotherapy may result in decreased efficacy and occurrence of additional side effects. Indeed, episodes of severe abdominal pain were exclusively observed in patients of the IIPCS-group of our study, whereas the rates of gastric and duodenal ulcers was similar in both groups. On the contrary, occurrence of hepatic arterial stenosis and thrombosis represented the major technical obstacle with the use of SIPCS. This may be explained by the fact that the catheter tip is fixed by suture within the hepatic artery with this technique, a fact that also makes interventional revisions impossible or at least problematic in most cases. As a result, there was a significantly higher rate of treatment-limiting vascular complications in the surgical than in the interventional group, resulting in a significant difference in port duration.

Another point of consideration is the difference in the application of folinic acid between both groups. In group A, a mixture of sodium folinate and 5-FU was administered, whereas a sequential application of calcium folinate and 5-FU was employed in group B. It seems very unlikely that this different practice has contributed to the difference in port complications, although no randomized comparisons on this topic are available either for the intraarterial nor the intravenous application [26,27]. Our results – at least – demonstrate that the combined use of sodium folinate and 5-FU in hepatic arterial infusion is not associated with a higher rate of device-related or vascular complications.

Comparing the baseline characteristics characteristics between both of our groups, the distribution of some items (elevated AP, number of pretreatments) indicate a rather unfavorable prognosis for the interventional group. This is underlined by the higher percentage of patients who primarily received a combination regimen, even though other factors – such as the introduction of novel drug combinations – may have contributed to this phenomenon. Due to these differences between both groups, it does not seem appropriate to compare response rates or other parameters of efficacy. However, it is important to mention that we did not observe unexpected complications in the scope of the implantation procedure or adjacent application of HAI in the IIPCS group. In this context, the absence of severe hepatotoxicity and biliary cirrhosis is in line with the results of other, larger studies on the regional use of 5-fluorouracil, where no respective episodes or treatment interruptions due to local side-effects have been reported [9,10]. Given the high performance rate of such devices on the one, and the fact that their implantation does not require laparatomy on the other hand, it appears that IIPCS enables the implementation of HAI even in colorectal cancer populations with a relevant percentage of heavily pretreated patients.

When interpreting these results, the non-randomised fashion of treatment allocation and the lack of comparisons with regard to oncological endpoints have to be considered. Indeed, we strictly focussed our phase II-trial on HAI provided through IIPCS on technical endpoints in a rather descriptive way, and aimed to work out at least an equivalence with the surgical approach within relatively heterogen collectives of patients with colorectal liver metastases. Being surprised about the clear result in favour of the interventional approach, the authors believe that data presented herein may be best understood as exploratory ones. They indicate a certain superiority of interventional port catheter implantation over the surgical approach with regard to both, technical failure rates and a broadened indication spectrum. Results seem to justify further evaluation of this topic and may serve as basis for the planning of future trials.

## Conclusion

HAI via interventionally implanted port catheters can be safely provided to a prospective collective of patients with colorectal liver metastases, including a relevant proportion of pre-treated individuals. It appears to offer technical advantages over the surgical approach.

## List of Abbrevations

HAI hepatic arterial infusion

IIPCS interventionally implanted port catheter systems

SIPCS interventionally implanted port catheter systems

FUDR floxuridine

5-FU 5-flourouracil

ECOG Eastern Cooperative oncology Group

DSA digital subtraction angiography

SPECT single photon emission computed tomography

Tc technetium

MAA macroaggregated albumine

FA folinic acid

WHO World Health Organisation

CT computed tomography

MRT magnetic resonance tomography

SPSS Statistical package for the Social Sciences

CEA carcinoembryogenic antigen

## Competing interests

*Bert Hildebrandt *has received honoriaria (below 10000 Euro each) from Pfizer, Roche, Sanofi-Avantis and Merck.

*Annett Nicolaou *has received honoriaria (below 10000 Euro each) from Roche.

*Birgit Bartels *has received honoriaria (below 10000 Euro each) from Pfizer, Roche, Sanofi-Aventis and Merck.

*Hanno Riess *has received honoraria (below 10000 Euro each) from Pfizer, Roche, Sanofi-Avantis and Merck; research funding from Roche (10–99000 Euro), Sanofi-Aventis (10–99000 Euro) and Merck (<10000 Euro); other remuneration (<10000 Euro) from Roche.

*Bernd Dörken *has served as consultant/advisor for the biotech company Cytonet, Weinheim, Germany, and holds stockings of the biotech company Develogen, Göttingen, Germany.

## Authors' contributions

BH contributed to the conception and design of the study, provision of patients, collection and assembly of data, data analysis and interpretation. He prescribed and administered regional chemotherapy. He has written the first draft of the manuscript and implemented the revisions of the co-authors.

MP contributed to provision of patients, collection and assembly of data, data analysis and interpretation, and manuscript writing. He performed the interventional implantation of hepatic arterial port catheters.

AN was involved into the provision of patients, collection and assembly of data, data analysis and interpretation. She prescribed and administered regional chemotherapy.

JML contributed to the provision of patients, and collection and assembly of data. He performed the surgical implantation of hepatic arterial port catheters.

JK contributed to the collection and assembly of data, data analysis and interpretation.

BB was involved into the collection and assembly of data, data analysis and interpretation.

AM was involved into the collection and assembly of data, data analysis and interpretation.

RF contributed to the conception and design of the study, provision of patients, collection and assembly of data.

PN performed the surgical implantation of hepatic arterial port catheters. He also contributed to the conception and design of the study and provision of patients.

HR contributed to the conception and design of the study, as well as, to the provision of patients. He prescribed and administered regional chemotherapy. He gave administrative support and was involved into manuscript writing.

BD contributed to the provision of patients, and the collection and assembly of data, gave administrative support, and was involved into manuscript writing.

JR was involved into the conception and design of the study, provision of patients, collection and assembly of data, data analysis and interpretation, and manuscript writing. He introduced and performed the interventional implantation of hepatic arterial port catheters.

All authors have revised the manuscript critically, and have given final approval of the version to be published.

## Pre-publication history

The pre-publication history for this paper can be accessed here:



## Figures and Tables

**Table 1:** Patients' characteristics

	**Group A: **Interventional	**Group B: **Surgical
Age (median/range)	60 (28–77)	59 (31–71)
Sex (f/m)	18/23	14/26
Primary tumour:		
colon/rectum	27/14	26/14
grading (G1/G2/G3/Gx)	1/26/12/2	0/29/10/1
Number of liver metastases:		
solitary/2–4/> 4	12/3/26	16/3/21
Occurrence of metastases		
synchron/metachron	19/22	24/16
Pre-treatment:		
Resection of primary	40	40
Hepatic resection/ablation	17	11
Adjuvant radiotherapy/chemotherapy radiochemotherapy	13	11
Palliative chemotherapy	17	9
more than 1 palliative pre-treatment *	9	0
Elevated CEA	29	32
Elevated Alkaline phosphatase *	30	16

* only significant values are indicated

**Table 2:** Treatment characteristics

	**Group A: **Interventional	**Group B: **Surgical	p-value*
**Intention of treatment:**			
"adjuvant" after resection/ablation	10	9**	
palliative	31	31	
**Type of first HAI – chemotherapy:**			
none (primary port failure)	0	2	
First-line FA/5-FU only	27	32	
First-line combination	14	6	0.046
Switched to 2nd-line HAI	16	12	
Switched to 3rd-line HAI	3	2	
Received HAI with MMC (any line)	8	7	
Received HAI with L-OHP (any line)	16	1	< 0.001
Received HAI and i.v. CPT-11 (any line)	9	0	< 0.001
**Complications and revisions**			
≥ 1 complication	26	30	
≥ 2 complications	9	11	
≥ 1 revision	23	4	< 0.001
≥ 1 thrombolytic therapy	7	11	
**Reason for termination of HAI:**			
regular/ongoing	12	4	0.029
disease progression	19	14	
port complication	7	20	< 0.001
chemotherapy-related toxicity	1	1	
withdrawal	2	0	

* only significant values are indicated

** 8 patients of group A and 7 patients of group B completed 6 courses of adjuvant treatment (see text for further details)

FA = folinic acid; 5-FU = 5-fluorouracil; MMC = mitomycin C; L-OHP = oxaliplatin; CPT-11 = irinotecan

**Table 3:** Overall port complications

	**GROUP A: Interventional**		**GROUP B: Surgical**		***p value***
**Number of pts./treatment courses**	**41/260**		**40/245**		

	***Abs.***	***%***	***Abs.***	***%***	

**Number of complications**	**39**	**100.0**	**45**	**100.0**	
***vascular***	**25**	**64.1**	**36**	**77.8**	***0.103***
Dislocation of catheter tip	8	20,5	3	6.7	0.061
Thrombosis (total)	7	17.9	10	22.2	0.627
Thrombosis (partial)/embolism	6	15.4	9	20.0	0.582
Stenosis	0	0.0	7	15.6	0.010
Dissection	3	7.7	6	13.3	0.404
Reflux	1	2.6	1	2.2	0.918
***non vascular***	**14**	**38.5**	**9**	**22.2**	***0.103***
Leakage/extravasation	4/1	12.8	3/1	8.9	0.561
Disconnection/rupture	4/1	12.8	0/1	2.2	0.060
Rotation of port chamber	0	0.0	2	4.4	0.183
Infection of port chamber	3	7.7	1	2.2	0.240
others	1	2.6	1	2.2	0.918

**Table 4:** Treatment-limiting port complications

	**GROUP A: Interventional (n = 41)**		**GROUP B: Surgical (n = 40)**		***p-value***
	***Abs.***	***%***	***Abs.***	***%***	

***vascular***	**5**	**12.2**	**15**	**37.5**	***0.008***
Thrombosis	5	12.2	9	22.5	*0.220*
Hepatic arterial stenosis	0	0	2	5.0	*0,147*
Dislocation of catheter tip	0	0	2	5.0	*0.147*
Reflux gastric/duodenal artery	0	0	1	2.5	*0.308*
Dissection hepatic artery	0	0	1	2.5	*0.308*
***non vascular***	**2**	**4.9**	**5**	**12.5**	***0.222***
Leakage/extravasation	0	0	3	7.5	*0.074*
Rotation of port chamber	0	0	1	2.5	*0.308*
Infection of port chamber	2	4.9	1	2.5	*0.571*
